# Mechanistic Insights in the Success of Fecal Microbiota Transplants for the Treatment of *Clostridium difficile* Infections

**DOI:** 10.3389/fmicb.2018.01242

**Published:** 2018-06-12

**Authors:** Amoe Baktash, Elisabeth M. Terveer, Romy D. Zwittink, Bastian V. H. Hornung, Jeroen Corver, Ed J. Kuijper, Wiep Klaas Smits

**Affiliations:** ^1^Clinical Microbiology Laboratory, Department of Medical Microbiology, Leiden University Medical Center, Leiden, Netherlands; ^2^Center for Microbiome Analyses and Therapeutics, Leiden University Medical Center, Leiden, Netherlands; ^3^Netherlands Donor Feces Bank, Leiden, Netherlands; ^4^Experimental Bacteriology, Department of Medical Microbiology, Leiden University Medical Center, Leiden, Netherlands

**Keywords:** fecal microbiota transplant, *Clostridium difficile* infection, short chain fatty acids, bile acids, sialic acids, antimicrobial peptides, bacteriocins, bacteriophages

## Abstract

Fecal microbiota transplantation has proven to be an effective treatment for infections with the gram-positive enteropathogen *Clostridium difficile*. Despite its effectiveness, the exact mechanisms that underlie its success are largely unclear. In this review, we highlight the pleiotropic effectors that are transferred during fecal microbiota transfer and relate this to the *C. difficile* lifecycle. In doing so, we show that it is likely that multiple factors contribute to the elimination of symptoms of *C. difficile* infections after fecal microbiota transplantation.

## Introduction

Fecal microbiota transplantation (FMT), the transfer of (processed) fecal material from healthy donors to patients, has been documented already over 1700 years ago for the treatment of gastrointestinal illness in humans. The Chinese scholar Ge Hong used fecal suspension administered orally to treat severe diarrhea; the 16th century scientist Li Shizhen referred to such suspensions as “yellow soup" (Zhang et al., 2012). Similarly, during World War II, soldiers stationed in North-Africa took up the practice of treating dysentery using camel dung, after observing locals do so (Lewin, 1999). Though in either case the rationale for treatment was unknown, anecdotal reports of successful treatment triggered a renewed scientific interest in the middle of the 20th century.

The first description of a fecal enema as treatment in modern medical literature dates from 1958, when four patients with pseudomembranous colitis were treated (Eiseman et al., 1958). At that time not identified yet as such, the major causative agent of pseudomembranous colitis is *Clostridium difficile*, a gram-positive, spore forming, obligate anaerobic bacterium (Hall and O’Toole, 1935; George et al., 1978; Larson et al., 1978; Smits et al., 2016). It was originally identified as part of the normal gut microbiota of healthy infants in 1935 (Hall and O’Toole, 1935) and was recently reclassified as *Clostridioides difficile*, based on phenotypic, chemotaxonomic and phylogenetic analyses (Lawson et al., 2016). 16S rRNA gene sequence analysis showed that the closest relative is *Clostridium mangenotii* (Lawson et al., 2016). Ground-breaking work in the 70’s established that *C. difficile* was a transmissible pathogen that produced toxin(s) capable of inducing gastrointestinal disease in various animals (Chang et al., 1978; Bartlett, 2009). *C. difficile* infection (CDI) is believed to be triggered by antibiotics which disrupt the normal microbiota enabling the outgrowth of toxin-producing *C. difficile* (Dethlefsen and Relman, 2011; Rea et al., 2011; Hensgens et al., 2012; Keller and Kuijper, 2015).

The first-line therapy of CDI according the European Society of Clinical Microbiology and Infectious Diseases (ESCMID) guideline is with the prodrug metronidazole, that is converted by anaerobic bacteria into nitroso radicals that exert an antimicrobial effect (Debast et al., 2014). However, metronidazole appears less effective compared with vancomycin and fidaxomicin in inducing initial cure (Johnson et al., 2014; Ooijevaar et al., 2018). As a result, vancomycin and fidaxomicin are recommended in the 2017 update of the guidelines of the Infectious Diseases Society of America and Society for Healthcare Epidemiology of America, even for mild and first occurrence CDI (McDonald et al., 2018). Nevertheless, the risk of recurrence after treatment within 8 weeks is 15–25% and rises to 40–65% in patients suffering from multiple recurrences (Fekety et al., 1997; Cornely et al., 2012; Johnson et al., 2014; Keller and Kuijper, 2015).

16S rRNA gene sequence analysis of gut microbiota of patients with an initial or recurrent CDI showed that patients with recurrent disease showed a highly variable bacterial composition in comparison with the normal predominance of Bacteroidetes and Firmicutes. Furthermore, patients with recurrent CDI showed lower species richness compared with patients with an initial episode of CDI patients and control subjects (Chang et al., 2008; Seekatz et al., 2016). This suggested that modulating microbiota composition could be key in the treatment of recurrent CDI.

The first randomized control trial using FMT to treat recurrent CDI (16 patients in the FMT arm versus 26 in the control arms) demonstrated a remarkable efficacy (van Nood et al., 2013), which was confirmed in multiple independent analyses. For instance, the meta-analysis by Quraishi and co-workers, that included seven randomized controlled trials (RCTs) and 30 case series, showed that FMT is more effective than vancomycin in resolving recurrent and refractory CDI with a relative risk of 0.23 and a clinical resolution of 92% (Quraishi et al., 2017). The meta-analysis by Moayyedi and co-workers included ten RCTs with a total of 657 patients with *C. difficile*-associated diarrhea and demonstrated that FMT was significantly more effective compared with placebo or vancomycin treatment, with a relative risk of 0.41 (Moayyedi et al., 2017). However, great heterogeneity exists among the included studies with respect to donor feces volume, FMT preparations, route of administration, pre-treatment and numbers of FMTs (Moayyedi et al., 2017; Quraishi et al., 2017; Terveer et al., 2018). After FMT, patients show an increase in microbiota diversity, reaching levels that are observed in healthy donors (van Nood et al., 2013; Fuentes et al., 2017). Taken together, the data show that for patients suffering from multiple recurrent CDI, FMT is a highly effective treatment (Ooijevaar et al., 2018) and FMT is now indicated in therapeutic guidelines for this group of patients (McDonald et al., 2018). Recommended treatment modalities and FMT procedures have been reviewed elsewhere (Cammarota et al., 2017; Terveer et al., 2017b; Woodworth et al., 2017; Panchal et al., 2018).

**FIGURE 1:**
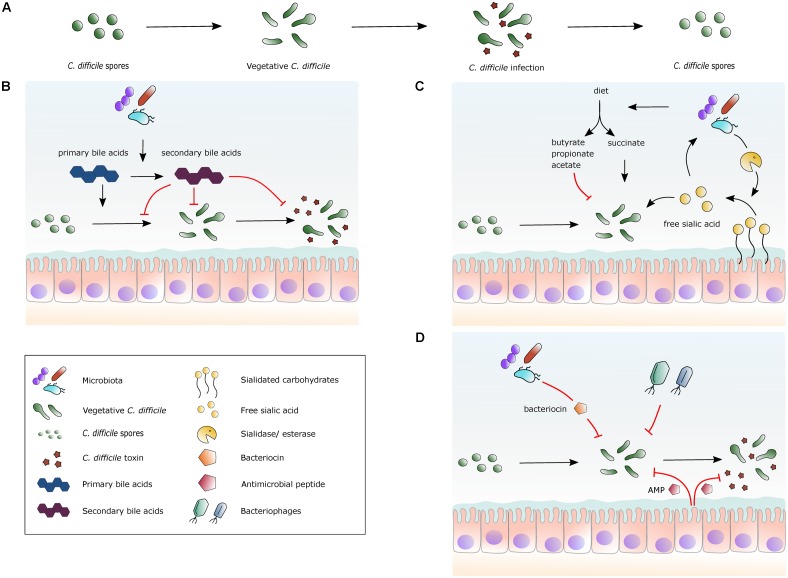
Selected mechanisms by which the gut microbiota, metabolites and peptides can affect *C. difficile*. **(A)** The lifecycle of *C. difficile.* Spores germinate into vegetative cells, which then proliferate and start to produce toxins. The lifecycle is completed by the formation of spores from vegetative cells again. **(B)** A simplified overview of the effects of bile acid metabolism on *C. difficile*. Secondary bile acids are formed from primary bile acids via 7α-dehydroxylation by gut bacteria. Primary bile acids promote germination of spores into vegetative cells, while secondary bile acids generally inhibit this process. Secondary bile acids can inhibit toxin production. **(C)** A simplified view of the effects of specific nutrients on *C. difficile*. Dietary fibers are fermented by the gut microbiota and short-chain fatty acids (SCFAs) are released. Succinate can stimulate *C. difficile* expansion, whereas other SCFAs potentially inhibit growth. Certain bacterial species in the gut cleave sialidated carbohydrates, releasing sialic acid that stimulates *C. difficile* expansion. **(D)** Schematic view of naturally occurring antimicrobial factors. The human gut has a high abundance of microbial species, some of which produce bacteriocins that are bacteriostatic or bactericidal for *C. difficile*. Certain bacteriophages present in the gut microbiota use *C. difficile* as a host. Antimicrobial peptides (AMP) are produced by host cells that inhibit *C. difficile* growth and inhibit toxin activity.

Multiple recurrent *C. difficile* infections remain the prime – and arguably only - example for which there is a consistent body of evidence for treatment by FMT and for which FMT is indicated as treatment strategy (Smits et al., 2016; McDonald et al., 2018; Ooijevaar et al., 2018). Yet, what is it in the donor material that results in the elimination of symptoms, and/or detectable presence of the pathogen? Fecal suspensions commonly used for FMT contain a plethora of abiotic and biotic factors. In this Review, we summarize our understanding of the possible active constituents in donor fecal material in relation to the *C. difficile* lifecycle (**Figure 1A**).

## Colonization Resistance

The first step in the establishment of an infection is colonization of a host with pathogenic *C. difficile* bacteria. Such colonization can result in asymptomatic carriership, or in disease (Eyre et al., 2013; Terveer et al., 2017a; Crobach et al., 2018). The onset of disease can be triggered through a disruption of the healthy microbiota due to exogenous factors such as antimicrobials (Britton and Young, 2014; Keller and Kuijper, 2015; Smits et al., 2016). In the early 1960’s, such antibiotic associated susceptibility to infection was also observed for *Salmonella enterica* (Bohnhoff and Miller, 1962). The hypothesis that a stable microbial community can prevent colonization and/or outgrowth of pathogens has become known as colonization resistance (Lawley and Walker, 2013). In its simplest view, FMT replenishes the microbial diversity that is lost after antimicrobial treatment and therefore confers colonization resistance against *C. difficile*. Though intuitively it is appealing that a dense microbial community exerts this effect, it should be noted that colonization resistance is as poorly defined as FMT. Microbial and immunological factors that play a role in colonization resistance (Lawley and Walker, 2013) are also likely to play a role in the success of FMT. Microbiota-immune interactions have been reviewed elsewhere (Belkaid and Hand, 2014; Khoruts and Sadowsky, 2016), and are not in the scope of this review. Here, we focus on the transferable components of a fecal suspension.

## Primary and Secondary Bile Acids

If we look beyond numbers, are there any specific mechanisms that might contribute to the efficacy of FMT? What small molecules have been shown to affect the lifecycle of *C. difficile*?

Spores are essential for the transmission of *C. difficile* between hosts and persistence in the (hospital) environment (Deakin et al., 2012; Paredes-Sabja et al., 2014). Upon passing the stomach, bile acids induce germination of *C. difficile* spores (Francis et al., 2013; Kochan et al., 2017) (**Figure 1B**).

Bile acids promote intestinal absorption and transport of lipids, nutrients and vitamins, but also serve a broad range of regulatory functions throughout the body (Chiang, 2009). The primary bile acids, cholic acid and chenodeoxycholic acid, are synthesized in the liver from cholesterol in a multi-step process and are conjugated to either glycine or taurine (Chiang, 2009). They are hydrophobic and conjugation increases their solubility (Staley et al., 2017). After food intake, bile acids are released into the duodenum and are reabsorbed by active transport in the distal ileum. This enterohepatic circulation has an overall recovery efficiency of ∼95% (Chiang, 2009). The secondary bile acids, deoxycholic acid and lithocholic acid, are formed via 7α-dehydroxylation by gut bacteria from bile acids that are not reabsorbed (Chiang, 2009). These bacteria are members of the Lachnospiraceae and Ruminococcaceae families and include *Clostridium scindens* (Weingarden et al., 2014; Buffie et al., 2015). Recirculated secondary bile acids are conjugated to glycine and taurine similar to primary bile acids (Chiang, 2009).

Bile acids can directly or indirectly influence the composition of the gut microbiota. Several studies demonstrated that bile acids have direct antimicrobial effects by damaging cell membranes and DNA through oxidative and pH stress (Kakiyama et al., 2013; Staley et al., 2017). There is a varying degree of bile resistance, tolerance and susceptibility even within single bacterial species (Staley et al., 2017). Bile acids can also regulate the gut community structure indirectly, as shown for cholic acid (Islam et al., 2011). Supplementation of the diet with cholic acid resulted in an increase of Firmicutes, a group that encompasses bacteria capable of 7α-dehydroxylating cholic acid, and decrease of Bacteroidetes (Islam et al., 2011; Ridlon et al., 2013).

Bile acids in the gastrointestinal tract affect the growth of *C. difficile* (**Figure 1B**). *In vitro*, primary bile acids generally stimulate germination, whereas secondary bile acids inhibit this process (Francis et al., 2013; Paredes-Sabja et al., 2014; Thanissery et al., 2017). The latter finding is recapitulated in a murine-derived model where physiologically relevant concentrations of primary and secondary bile acids where tested (Theriot and Young, 2015). In this model, *C. difficile* was able to germinate and grow in ileal an cecal content when secondary bile acids were depleted, demonstrating that secondary bile acids inhibit spore germination and growth (Theriot and Young, 2015).

Patients with CDI have an altered fecal bile acid composition in the colon. For instance, secondary bile acids were higher in fecal samples from controls compared to patients with CDI, and primary bile acids were elevated in patients with recurrent disease compared to those experiencing a first episode of CDI (Allegretti et al., 2016).

Fecal microbiota transplantation treatment has a significant effect on the bile acid composition. Pre-FMT fecal samples of CDI patients show a shift in the balance between primary bile acids and secondary bile acids, almost completely toward the former, whereas post-FMT fecal samples contain predominantly secondary bile acids, similar to donor samples (Weingarden et al., 2014). A combination of pre-FMT bile acids induced germination of *C. difficile in vitro*, whereas the post-FMT combination did not. Likewise, pre-FMT bile acids did not affect the vegetative growth of *C. difficile*, but bile acids from post-FMT feces significantly reduced growth (Weingarden et al., 2016b). Differences in bile acid composition are likely the result of microbiota-dependent differences in bile acid conversion. Indeed, the 7α-dehydroxylating intestinal bacterium *C. scindens* was associated with resistance to *C. difficile* infection (Buffie et al., 2015), and a simplified 12-species “oligo-mouse microbiota”(Oligo-MM^12^) that represents the major murine intestinal bacterial phyla, but is 7α-dehydroxylation deficient, fails to confer resistance to CDI (Studer et al., 2016). Supplementation of the mice with *C. scindens* normalized the intestinal bile acid composition and impacted early colonization with *C. difficile*, but was insufficient to prevent pathogenesis (Studer et al., 2016). It is hypothesized that antibiotics might reduce the amount of secondary bile acids by eliminating bile acid converting bacteria, thereby increasing the propensity for CDI (Weingarden et al., 2014).

However, it is important to note that a model where primary bile acids strictly promote and secondary bile acids inhibit *C. difficile* germination and vegetation might be oversimplified. Physiological concentrations of the primary bile acids taurocholic acid, cholic acid and higher concentrations of chenodeoxycholic acid induced germination of spores, whereas physiological concentrations of chenodeoxycholic acid did not (Weingarden et al., 2016b) or even inhibited spore germination (Sorg and Sonenshein, 2009, 2010; Theriot and Young, 2015). The secondary bile acids deoxycholic acid and litocholic acid did not induce germination of spores (Weingarden et al., 2016b), in contrast to other studies which have shown that deoxycholic acid can be a germinant for *C. difficile* (Wilson, 1983; Sorg and Sonenshein, 2008).

Interestingly, recent work suggests that bile acids or bile acid-derived molecules may also influence toxin-dependent effects. In a small molecule screen, methyl cholate – but not cholate – was identified as a compound that inhibits auto-processing and receptor binding (Tam et al., 2015), leading to a reduction of toxin-dependent epithelial damage. Deoxycholate (0.02%) was found to inhibit toxin levels for some, but not all *C. difficile* strains *in vitro* (Thanissery et al., 2017).

Collectively, the data above suggest that bile acids may be an important component of an FMT (Weingarden et al., 2014) and that modulation of bile acid composition may be a viable therapeutic intervention (Buffie et al., 2015; Studer et al., 2016): FMT may introduce bacteria that affect bile acid composition and subsequently *C. difficile* germination and growth, or bile acids already present in the donor material may directly influence these processes. In support of the latter, it was reported that oral ursodeoxycholic acid (a secondary bile acid) was successfully used in conjunction with vancomycin to treat a patient with recurrent CDI pouchitis (Weingarden et al., 2016a). Both mechanisms, however, may not be mutually exclusive.

## Carbohydrates and Other Nutrients

Upon germination, vegetative cells use the nutrients available in the gut to proliferate. The nutritional requirements and capabilities of *C. difficile* have been extensively studied (Hafiz and Oakley, 1976; Karasawa et al., 1995; Scaria et al., 2014), but our understanding of the nutrient availability in the gut lumen in relation to *C. difficile* infection is limited. A metabolomic analysis showed that colonization with *C. difficile* leads to shifts in detectable metabolites and underscored the importance of amino acids and other nutrient availability for *C. difficile* colonization and pathogenesis (Fletcher et al., 2018).

In relation to CDI, some of the best characterized effects concern the liberation of sialic acid (**Figure 1C**). The mammalian host is protected from direct interaction with gut-dwelling microbes due to a physical barrier of heavily glycosylated mucus components produced by specialized cells in the intestinal mucosa. The mucus consists of an inner layer and a “loose” outer layer (Atuma et al., 2001). The outer layer can act as an important source of nutrition for bacteria capable of digesting its carbohydrate chains (Marcobal et al., 2013; Robinson et al., 2017). These carbohydrate chains are often capped with sialic acid, a 9-carbon-backbone monosaccharide that protects them from enzymatic reactions of exo- and endoglycosidases. Certain intestinal bacteria are able to remove sialic acids by expressing sialidases (Marcobal et al., 2011; Robinson et al., 2017). To protect sialic acids from these enzymatic actions they are modified through O-acetylation. This modification takes place in the inner mucus layer at the 7-O position of the sialic acid. As the mucus matures and moves to the outer layer the acetylation moves from the 7-O to the 9-O position. Some intestinal bacteria encode O-acetyl esterases that can remove the acetyl group, thus giving access to the sialidases that can remove the sialic acids. The O-acetyl esterases can only remove 9-O- acetyl groups, but not 7-O- acetyl groups (Robinson et al., 2017). Thus, the inner mucus layer is always protected, whereas the outer layer can be a carbon source for intestinal bacteria. Not all bacterial species contain all enzymes required for digestion of complex carbohydrates. For example, *Bacteroides fragilis* encodes both sialidase and O-acetyl esterase activity, whereas *Bacteroides thetaiotaomicron* only has esterase activity. Bacteria that lack sialidases and esterases themselves can take advantage of the enzymatic activities provided by the other members of the microbiota to access nutrients and gain access to the epithelium (Marcobal et al., 2013; Robinson et al., 2017).

Antibiotic treatment can alter the balance in the mucolytic activities; elimination of species that efficiently scavenge sialic acid allows antibiotic-resistant pathogens to profit from the free sugars and expand rapidly as has been shown for both *Salmonella typhimurium* and *C. difficile* (Ng et al., 2013). In gnotobiotic mice colonized with sialidase-producing *B. thetaiotaomicron*, or by exogenous dietary administration of free sialic acid, a rapid expansion of the enteropathogens was observed. Colonization by a sialidase-deficient mutant of *B. thetaiotaomicron* reduced the levels of free sialic acid and impaired expansion of *C. difficile* (Ng et al., 2013). In an *in vitro* model it was also observed that other members of the microbiota compete more efficiently than *C. difficile* for monomeric glucose, N-acetylglucosamine and N-acetylneuraminic acid, in addition to sialic acid (Wilson and Perini, 1988).

Dietary carbon sources have also been implicated in *C. difficile* infection. Nosocomial outbreaks and sustained high infection rates in the community can frequently be attributed to the epidemic PCR ribotypes 027 and 078, respectively (Goorhuis et al., 2008; Bauer et al., 2011; He et al., 2013; Smits et al., 2016). Recently, it has been shown that both these PCR ribotypes can use low concentrations of trehalose more efficiently as a carbon source than non-epidemic types (Collins et al., 2018). In 027 strains this is the result of a point mutation in the trehalose repressor TreR that leads to stronger derepression in the presence of trehalose. Strains of PCR ribotype 078 appear to have acquired an extra gene cluster encoding a trehalose importer. The introduction of trehalose into the food chain might have preceded the expansion of the epidemic types, suggesting a causative effect (Collins et al., 2018).

Besides affecting growth, nutrients are also known to affect expression levels of the main virulence factors of *C. difficile*, the major clostridial toxins A (TcdA) and B (TcdB) (Bouillaut et al., 2015; Martin-Verstraete et al., 2016; Smits et al., 2016). For instance, the addition of glucose to *C. difficile* growth medium drastically reduces toxin gene expression (Dupuy and Sonenshein, 1998; Oliveira Paiva et al., 2016) and amino acids can both negatively and positively affect toxin levels (Yamakawa et al., 1994; Karlsson et al., 1999). In line with the effects observed for glucose, ethanolamine delays toxin production in *C. difficile*, in contrast to other gut pathogens that use ethanolamine as a virulence-inducing signal (Nawrocki et al., 2018). Ethanolamine is a breakdown product of membrane-derived phosphatidylethanolamine, which is abundant in the gut and is increased during inflammatory responses. Mutants of *C. difficile* that cannot utilize ethanolamine show enhanced virulence in a hamster model of infection (Nawrocki et al., 2018), that is exquisitely sensitive to levels of toxin A and B (Bakker et al., 2014).

It should be noted that most of these studies were performed *in vitro*, and it is largely unclear how the levels used in these experiments relate to luminal levels *in vivo*. Nevertheless, there is some recent evidence that dietary interventions can be relevant for CDI. Defined nutrient diets, in particular low protein diets, were found to increase survival of mice and reduce disease severity (Moore et al., 2015). Also, *C. difficile* burden in mice with humanized microbiota could be suppressed when the mice were fed with microbiota accessible carbohydrates found in dietary plant polysaccharides, whereas mice with diets deficient for such carbohydrates show a persistent infection (Hryckowian et al., 2018).

The effects of antimicrobials, nutrient availability, sensing nutritional status and toxin production by *C. difficile* come together in a recently formulated model (Hryckowian et al., 2017). It was suggested that *C. difficile* expansion following antibiotic treatment initially occurs without toxin production, possibly due to the presence of readily metabolizable nutrient sources such as glucose and sialic acid. After the expansion, *C. difficile* may switch to toxin production, leading to a favorable state of inflammation to inhibit competitors.

Considering the above, it is conceivable that the nutrient or carbohydrate availability in donor material affects the efficacy of FMT, either by affecting the (re)growth of *C. difficile* or by suppressing toxin expression and thereby reducing symptoms. However, these possible effects have not been systematically investigated to date and further studies are required to determine if nutrient variables should be monitored or adjusted in donor material.

## Short and Medium Chain Fatty Acids

Above, we discussed the relevance of nutrient levels for the growth and pathogenesis of *C. difficile*. Next, we want to extend this, by focusing on a particular class of carbohydrates: the short and medium chain fatty acids - monocarboxylic acids with a carbohydrate chain length of 1 to 12 carbon atoms (Schonfeld and Wojtczak, 2016) (**Figure 1C**). These compounds have gained interest in the context of gastrointestinal disease due to their immune-modulatory and antimicrobial effects (Maslowski et al., 2009).

Short-chain fatty acids (SCFAs; chain length 1–6), are the main products of dietary fibers that are fermented by anaerobic gut bacteria and serve as substrates for energy metabolism (Roediger, 1980; den Besten et al., 2013). The most abundant SCFAs are acetate, propionate and butyrate (den Besten et al., 2013; Schonfeld and Wojtczak, 2016). Other common end-products of primary fermenters are the organic acids succinate and lactate, which are metabolized by other bacteria (Ferreyra et al., 2014). In mice, SCFAs bind G-protein coupled receptor 43 (GPR43) which results in resolution of inflammatory responses providing a link between immune and inflammatory responses, diet and the metabolism of gut bacteria (Maslowski et al., 2009). Many SCFAs can be associated with an improvement of gut barrier function and also possess anti-inflammatory properties (Tedelind et al., 2007; Chen et al., 2017).

*C. difficile* metabolizes succinate to butyrate, particularly under antibiotic treatment or chemically induced diarrhea that results in an abundance of succinate and a reduction of acetate and butyrate (Ferreyra et al., 2014). A mutant *C. difficile*, deficient in succinate utilization, and therefore butyrate production, displays attenuated growth under both these conditions (Ferreyra et al., 2014). Though succinate is generally not detected in a healthy human gut, it was found that succinate accumulates in pigs suffering from antibiotic associated diarrhea (Tsukahara and Ushida, 2002). Succinate therefore may be depleted by cross-feeding (i.e., taken up and metabolized) and antibiotic use may perturb these interactions (Ferreyra et al., 2014).

Several studies have reported differences in SCFA producing bacteria and/or SCFA levels between healthy subjects and CDI patients: 16S rRNA gene analysis shows that butyrate producers, such as Ruminococcaceae and Lachnospiraceae families, are significantly reduced in CDI patients compared to controls (Antharam et al., 2013; Song et al., 2013; Ling et al., 2014; Lamendella et al., 2018). Concomitantly, increased levels of succinate, lactate, formate producers such as *Bacteroides* spp. were found (Antharam et al., 2013; Ling et al., 2014).

Short-chain fatty acid levels also appear to correlate with colonization resistance against *C. difficile* in certain cases. Mice treated for 2 weeks with cefoperazone are susceptible to colonization by *C. difficile* and metabolomics analyses showed reduced levels of acetate, propionate and butyrate compared to non-treated mice (Theriot et al., 2014). Six weeks after cessation of the antimicrobial treatment, colonization resistance was fully restored and SCFA levels recovered, though not to the levels observed in non-treated mice (Theriot et al., 2014). In earlier work, colonization resistance in hamsters was also found to coincide with high levels of SCFAs, that inhibit *C. difficile* growth (Rolfe, 1984). Cecal SCFA levels increase in hamsters from day 1, and reach a maximum at 19 days. Hamsters are susceptible to *C. difficile* colonization between days 4 and 15 and levels of SCFA corresponding to day 16 onward are bactericidal to *C. difficile* cultures *in vitro* (Rolfe, 1984).

The correlations between CDI and SCFAs have triggered investigations to modulate CDI development and progression through dietary interventions. Non-digestible oligosaccharides (dietary fiber) are carbohydrates resistant to the effects of gastrointestinal enzymes, but can be fermented to SCFAs by members of the colonic microbiota. Indeed, the addition of fiber to an *in vitro* model using fecal inoculums from pigs leads to high levels of acetate, propionate and butyrate that correlates with the expansion of anaerobic bacteria (May et al., 1994). In an *in vitro* model that simulates the conditions of the proximal part of the large intestine, the introduction of *C. difficile* leads to a suppression of propionate production and an increase in the branched-chain fatty acids isobutyrate and isovalerate (van Nuenen et al., 2003). The addition of inulin, a non-digestible oligosaccharide, reversed this effect and increased the total amount of SCFAs by 50% (van Nuenen et al., 2003). In another experiment, supplementation of *in vitro* cultures with different oligosaccharides increased SCFA production and did not result in detectable *C. difficile* toxin (Hopkins and Macfarlane, 2003). However, this was not the case during clindamycin treatment, where supplementation led to a reduction in SCFAs and conditions conducive for toxin production by *C. difficile*. (Hopkins and Macfarlane, 2003)

Despite the evidence presented above, a role for SCFA in control of CDI is not undisputed. No correlation was found between a qualitative SCFA profile of fecal emulsion and its ability to inhibit the growth of *C. difficile in vitro* (Borriello and Barclay, 1986). Furthermore, in a gnotobiotic mouse model, SCFAs alone did not affect colonization by *C. difficile* (Su et al., 1987). More recently, partial restoration of colonization resistance was reported when germ free mice were pre-colonized with *Lachnospiraceae*, but not with *E. coli* (Reeves et al., 2012). Though many *Lachnospiraceae* are SCFA producers (Scott et al., 2014), no association between SCFA production and *C. difficile* colonization levels was found (Reeves et al., 2012). Importantly, neither a fecal filtrate (containing SCFA), nor SCFAs were able to clear a *C. difficile* infection in mice (Lawley et al., 2012).

Medium chain fatty acids (MCFAs; with chain length 7–12) are predominantly derived from triglycerides and phospholipids that are ingested as part of plant oils and milk products (Schonfeld and Wojtczak, 2016). MCFAs can have antimicrobial activity, and modest activity of the 12-carbon lauric acid – a constituent of virgin coconut oil - against *C. difficile* has been reported (Shilling et al., 2013; Yang et al., 2017). Exposure of *C. difficile* cells to lauric acid leads to oxidative damage and cell lysis at higher concentrations (Shilling et al., 2013; Yang et al., 2017). Interestingly, orogastric pre-treatment with lauric acid decreased symptoms in a mouse model of CDI. However, at present it is unclear if these effects are due to an antimicrobial effect on *C. difficile* cells or due to an effect on the host (Yang et al., 2017).

In total, a causal relationship between SCFA and MCFA levels and *C. difficile* pathogenesis remains doubtful. Though a role for these compounds in donor material seems plausible, we expect that an analysis of fatty acid composition will potentially only be informative when placed in the context of the microbial composition and other variables.

## Biological Warfare

Other species of the microbial community not only interfere with *C. difficile* by competing for, or cross-feeding on, nutrients. Many bacterial species are known to produce antimicrobial compounds that allow the elimination of competitors (Tracanna et al., 2017). It is conceivable that the microbiota transferred by FMT produces specific bacteriocins that kill *C. difficile* (Khoruts and Sadowsky, 2016) (**Figure 1D**). Bacteriocins are proteinaceous antimicrobial compounds and are synthesized in many bacteria by ribosomes during translation (Le Lay et al., 2016). Their inhibitory activity may be small-spectrum or broad-spectrum and there are bactericidal and bacteriostatic bacteriocins. In general, their activity is directed against bacteria that are phylogenetically close to the producing bacteria (Tagg et al., 1976). Some of the antimicrobial peptides that target *C. difficile* are discussed below.

Thuricin CD is a two-component bacteriocin, consisting of peptides Trn-α and Trn-β, produced by *Bacillus thuringiensis* DPC 6431, a bacterium derived from a human fecal sample (Rea et al., 2010; Sit et al., 2011). It displays a narrow spectrum of activity against mainly spore-forming gram-positive bacteria of the class Clostridia and Bacilli. All *C. difficile* isolates tested were sensitive to supernatants of *B. thuringiensis* DPC 6431, including the epidemic PCR ribotype 027 NAP1. In an *ex vivo* distal colon model, thuricin CD showed similar efficacy as metronidazole (Rea et al., 2010) and vancomycin (Rea et al., 2011).

Nisin is a bacteriocin with broad antimicrobial activity against a wide range of gram-positive bacteria (Lubelski et al., 2008). It can be isolated from the known gut residents such as *Blautia obeum* A2-162 (Hatziioanou et al., 2017) and species that can proliferate in conditions that resemble the gut, including *Lactococcus lactis* UL719 (Le Lay et al., 2015). *In vitro* studies show that nisin can inhibit spore germination and vegetative growth of *C. difficile* (Le Lay et al., 2016). In a human colon model, *L. lactis* UL719 did not significantly alter the microbiota composition and was not effective against *C. difficile*, likely because nisin produced *in vivo* does not reach inhibitory levels (Le Lay et al., 2015).

*Lactobacillus reuteri* carrying the *pocR* gene converts glycerol to reuterin, a broad-spectrum antimicrobial compound (Spinler et al., 2017). Reuterin induces oxidative stress, most likely by modifying thiol groups (Schaefer et al., 2010). Reuterin production increases in *L. reuteri* upon interaction with other bacteria (Schaefer et al., 2010). Co-delivery of *L. reuteri* and glycerol was effective and decreased the abundance of *C. difficile* relative to the bacterial load in fecal mini-bioreactor arrays pre-treated with antibiotics, without significantly affecting the microbial composition, whereas reuterin, *L. reuteri* or glycerol alone did not achieve this effect. This indicates that viable *L. reuteri*, substrate and active reuterin production is required for growth inhibition of *C. difficile* (Spinler et al., 2017).

Other antimicrobial compounds with activity against *C. difficile*, alone or in combination, include enterococcal durancin (Hanchi et al., 2017), formicin from *Bacillus paralicheniformis* (Collins et al., 2016), microbisporicin from the actinomycete *Microbispora* (Castiglione et al., 2008), and the lactococcal lacticin 3147 (Rea et al., 2007).

The potential for members of the microbiota to inhibit *C. difficile* is underscored by the fact that the clinically used therapeutic fidaxomicin, as well as the pre-clinical compound surotomycin were identified as natural products (Kociolek and Gerding, 2016). Fidaxomicin is produced by the actinomycete *Dactylosporangium aurantiacum* subspecies *hamdenesis* as a byproduct of fermentation. It prevents transcription by inhibiting bacterial RNA polymerase and is bactericidal against *C. difficile*. Fidaxomicin is non-inferior to oral vancomycin in clinical response and superior to oral vancomycin in reducing recurrent CDI (Louie et al., 2011; Cornely et al., 2012). Surotomycin, a cyclic lipopeptide antibiotic with a core derived from *Streptomyces roseosporus*, acts on the membrane stability of *C. difficile*, both in logarithmic and stationary phases (Knight-Connoni et al., 2016). It has a low oral absorption that allows high concentrations in the gastrointestinal tract to be achieved. Despite promising phase II results, phase III studies found the compound to be inferior to vancomycin (Boix et al., 2017). Both fidaxomicin and surotomycin claim a minimal impact on the gut microbiota (Kociolek and Gerding, 2016).

In addition to antimicrobial compounds produced by other members of the microbiota, the lumen of the colon also contains host-defense molecules that might play a role in CDI progression and pathogenesis (**Figure 1D**). These include defensins, cathelicidins such as LL-37 and lysozyme (McQuade et al., 2012). Defensins (α-defensins: HD5-6 and HNP1-4; β-defensins: HBD1-4) are small cationic peptides derived from intestinal Paneth cells, neutrophils or epithelial cells (Nuding et al., 2014). The human cathelicidin LL-37 is released upon cleavage of the precursor hCAP-18, which is produced in epithelial cells and some immune cells (Nuding et al., 2014). Lysozyme hydrolyses peptidoglycan bonds between N-acetyl-glucosamine and N-acetylmuramic acid and as a result the bacteria become sensitive to lysis (Chai et al., 2015).

Defensins and LL-37 were tested alone or in combination with antibiotics (tigecycline, moxifloxacin, piperacillin-tazobactam, and meropenem) against toxigenic and non-toxigenic *C. difficile* strains (Nuding et al., 2014). The antimicrobial peptides alone demonstrated some antimicrobial activity against *C. difficile*, but when combined, an additive effect was observed (Nuding et al., 2014). Certain treatments resulted in increased toxin release, presumably due to lysis of *C. difficile* (Nuding et al., 2014). This suggests that antimicrobial peptides alone or in combination with antibiotics not only exert a positive effect on CDI (clearance of *C. difficile*), but could potentially also worsen symptoms (by inducing more inflammation). Interestingly, α-defensins (HNP-1, HNP-3, and HD-5) appear to inhibit toxin B, but not toxin A, activity. The inhibition was dose dependent and reversible. β-defensins and LL-37 did not inhibit toxin A or toxin B (Giesemann et al., 2008). Similarly, LL-37, or the murine homolog cRAMP, can modulate the inflammatory response to toxins in a mouse model of infection (Hing et al., 2013). Lysozyme can act synergistically with bacterial antimicrobial peptides, as shown for nisin (Chai et al., 2015).

The effectiveness of human defense molecules might depend on the *C. difficile* isolate as variation in susceptibility to LL-37 was observed between *C. difficile* strains, with epidemic ribotype 027 strains being less susceptible than non-RT 027 strains (McQuade et al., 2012). *C. difficile* is capable of mounting a response to antimicrobial peptides and lysozyme (McBride and Sonenshein, 2011a,b; Hastie et al., 2014), but the contribution of these mechanisms to the observed variability is unknown.

There is very limited data on the role that defense molecules might play in FMT. A study describing the colonoscopic FMT for the treatment of CDI reported that LL-37 levels in plasma were significantly increased 3 months post-FMT, compared to pre-FMT and 3-weeks post-FMT (Konturek et al., 2016). As cathelicidins are important in preventing intestinal barrier dysfunction, this was taken as an indication of recovery from CDI-damage (Konturek et al., 2016). It does indicate however, that healthy subjects likely demonstrate higher levels of LL-37 than CDI patients. It has been demonstrated that endogenous levels of cathelicidins are likely insufficient to maintain barrier function, but that high levels of exogenously provided LL-37 or cRAMP can reduce CDI symptoms in a mouse model (Hing et al., 2013).

Overall, we believe that antimicrobial peptides – whether derived from the microbiota or the host – have the potential to contribute to the efficacy of FMT. However, a clear correlation between FMT success and levels of these compounds in donor material remains to be established.

## Bacteriophages

Above we discussed how inter-bacterial competition and host-defense can modulate the gut microbiota. However, another level of complexity exists in the form of bacteriophages (**Figure 1D**).

Bacteriophages are viruses, composed of proteins that encapsulate a DNA or RNA genome, and replicate within bacteria and archaea (Salmond and Fineran, 2015; Ofir and Sorek, 2018). Binding of bacteriophages to specific receptors on the bacterial cell determines their host range (Dowah and Clokie, 2018). Lytic phages infect, multiply in and lyse bacterial cells. In contrast, lysogenic phages can integrate into host DNA and replicate along with it, or become established as a plasmid without immediate lysis of the host (Salmond and Fineran, 2015; Ofir and Sorek, 2018). Phage exposure can not only affect the composition of the microbiota, but may also alter different phenotypes, including virulence and biofilm formation (Mirzaei and Maurice, 2017; Fernandez et al., 2018).

Prophages and phages have been found in pathogenic clostridia (Hargreaves and Clokie, 2014; Fortier, 2017). For instance, neurotoxin and α-toxin (TcnA) of *Clostridium botulinum* types C and D and *Clostridium novyi*, respectively, are encoded on prophages. In *C. difficile*, prophage carriage is high and one particular strain has been found to contain binary toxin on a prophage (Hargreaves et al., 2013; Fortier, 2017; Riedel et al., 2017). Phages known to infect *C. difficile* belong to the order of Caudovirales and, more specifically, the families Myoviridae and Siphoviridae (Hargreaves and Clokie, 2015; Ramirez-Vargas et al., 2018). As observed for other viruses, (pro)phages have been found to alter (virulence) gene expression in *C. difficile* (Goh et al., 2005; Govind et al., 2009; Sekulovic et al., 2011; Hargreaves et al., 2014b; Sekulovic and Fortier, 2015). CRISPR (clustered regularly interspaced short palindromic repeats)-Cas (CRISPR-associated) adaptive immune systems are widespread among bacteria and archaea. Recent studies have shown that these systems have minimal long-term evolutionary effects in limiting horizontal gene transfer (Pawluk et al., 2018). *C. difficile* contains a CRISPR-Cas system that provides defense against plasmid DNA and bacteriophages, as has been demonstrated in reference strain 630 and the epidemic ribotype 027 strain R20291 (Hargreaves et al., 2014a; Boudry et al., 2015; Andersen et al., 2016).

Phages are induced during CDI as sterile filtrates of CDI patients contained multiple phages (Meessen-Pinard et al., 2012). CDI patients display a higher abundance of Caudovirales than healthy household controls, and lower diversity, richness and evenness (Zuo et al., 2018). Moreover, treatment with antibiotics such as quinolones induced prophage mobility *in vitro* (Meessen-Pinard et al., 2012), as has also been observed by others (Modi et al., 2013). It therefore seems likely that both the virulence potential and horizontal gene transfer could be affected under conditions that sustain *C. difficile* expansion and pathogenesis.

Phages can be employed to treat bacterial infectious diseases (Abedon, 2017). At a time of growing antibiotic resistance in bacteria and the resulting restrictions on the use of antibiotics, bacteriophages can provide an alternative means of eliminating pathogens. Is it possible to use bacteriophages to treat CDI? Phage ΦCD27 demonstrates a significant reduction in *C. difficile* cell numbers and toxin production in an *in vitro* human colon model of CDI, without major effects on other members of the microbiota (Meader et al., 2010; Meader et al., 2013). Combinations of phage in batch cultures resulted in a 6-log reduction of *C. difficile* ribotype 014/020 (Nale et al., 2018), a type commonly identified amongst clinical isolates (Bauer et al., 2011), and were found to be able to penetrate established biofilms (Nale et al., 2016a). Though resistance to individual phages can be problematic, the resistant *C. difficile* remain susceptible to other phages (Nale et al., 2016b). Results obtained in animal models largely mirror the *in vitro* results. In hamsters, the administration of optimized phage cocktails resulted in reduced *C. difficile* colonization and recovery of free phage from the lumen of the cecum and the colon; moreover, pre-treatment with a phage cocktail delayed the onset of disease (Nale et al., 2016b). In a wax moth (*Galleria mellonella*) model, a phage cocktail was effective alone, or as adjunct therapy to vancomycin (Nale et al., 2016a). Besides live phages, phage-derived proteins are also explored as alternatives for treatment (Mayer et al., 2011; Kirk et al., 2017). Notably, phages demonstrate increased virulence toward bacteria in the presence of eukaryotic cells (Shan et al., 2018).

There are a limited number of studies that investigate phages during FMT treatment. A case study of a recurrent CDI patient demonstrated donor-similar characteristics of the virome following FMT (Broecker et al., 2017). Similarly, after FMT of a single healthy donor to three ulcerative colitis patients, donor-similar viral sequences were readily detected (Chehoud et al., 2016). Interestingly, temperate phages such as Siphoviridae were more likely to be transferred, suggesting that transfer or establishment may be dependent on lysogeny (Chehoud et al., 2016). An ultra-deep metagenomic sequencing study of viral transfer during FMT analyzed longitudinal samples of 9 CDI patients receiving FMT, and 5 receiving vancomycin treatment (Zuo et al., 2018). FMT treatment decreased the absolute abundance of Caudovirales, and treatment response correlated with the presence of donor-derived phage among those that were detected (Zuo et al., 2018). This suggests a role for Caudovirales bacteriophage in the efficacy of FMT. This is supported by a recent study where sterile (bacteria-free) fecal filtrates were transferred to 5 patients with symptomatic chronic-relapsing CDI (Ott et al., 2017). The transfer of the filtrates, that showed a complex signature of bacteriophages, prevented recurrent CDI in all 5 patients for a minimum period of 6 months.

Taken together, it appears that the concept of CDI-predisposing dysbiosis, as a result of for instance antimicrobial treatment, could be extended to include bacteriophage and that phage therapy could be useful in the treatment of disease (Manrique et al., 2017).

## Others

So far, this review has focused on variables with a strong biotic component. However, abiotic factors, including metal availability, are known to affect infectious diseases (Fischer Walker and Black, 2004). Several secreted and surface-exposed proteins of *C. difficile* contain zinc as a co-factor, prompting an investigation of the effects of dietary zinc in a mouse model of CDI (Zackular et al., 2016). It was reported that mice on a high-zinc diet displayed a reduced diversity of their gut microbiota, and a concomitant reduction in threshold of antibiotics needed to induce CDI. Furthermore, consistent with a role in Toxin A auto-processing (Chumbler et al., 2016), CDI was exacerbated by an increased toxin activity in mice on a high-zinc diet. The mechanisms by which excess Zn alters the structure of the gut microbiota is unknown, but the authors postulate that it is a combination of several factors and consider metal toxicity to specific bacterial species as one of them (Zackular and Skaar, 2018). Human calprotectin is a zinc binding protein with broad activity against bacterial pathogens (Zackular et al., 2015). Consistent with a role in the innate immune response against *C. difficile*, calprotectin levels correlate with adverse outcome in CDI (Rao et al., 2016) and calprotectin-deficient mice show more severe CDI (Zackular et al., 2016).

Other metals have also been implicated in CDI. Divalent calcium ions are important for efficient germination of *C. difficile* and patients with inefficient calcium adsorption have a predisposition for CDI (Kochan et al., 2017).

Though the effects of levels of metals and metal-binding proteins such as calprotectin have not been systematically investigated, there is an intriguing possibility of modulating the efficacy of FMT by adjusting metal availability.

## Conclusion

Many studies have shown that FMT is an effective treatment in patients with recurrent CDI (van Nood et al., 2013; Moayyedi et al., 2017; Quraishi et al., 2017; Terveer et al., 2017a; Ooijevaar et al., 2018). In the future, FMT may find other applications beyond *C. difficile* infections (Bakker and Nieuwdorp, 2017). However, since systematic investigations of FMT are relatively new, the mechanisms underlying the efficacy remain largely unknown. We expect that different mechanisms may play a role for other diseases than CDI.

We believe that it is unlikely that a single factor is responsible for the efficacy of FMT; the possible mechanisms examined in this review indicate that although individual variables evidently can modulate CDI, the mechanism of FMT is likely multifactorial. Indeed, FMT was found to restore both SCFA levels and bile acid metabolism (Seekatz et al., 2018). It should be noted that the gut microbiota is both influenced by, and influences, levels of compounds such as bile acids, short chain fatty acids and nutrients, complicating analyses of cause and effect (Seekatz and Young, 2018).

Current evidence suggests that part of the success of FMT can be attributed to the reconstitution of a robust and diverse microbiota of the gut (Lawley et al., 2012; van Nood et al., 2013). These findings were challenged, however, by the recent report that sterile filtrates were effective in treating recurrent CDI (Ott et al., 2017). It should be noted that these results have not been replicated yet in a randomized control trial, and are in contradiction with a study in mice that shows that transfer of buffer, fecal filtrate, a solution of short chain fatty acids or a single bacterial strain is unable to clear a *C. difficile* infection (Lawley et al., 2012). Perhaps non-bacterial components of a fecal filtrate are capable of transiently suppressing *C. difficile* growth, while allowing regrowth of a seeding population of a diverse microbiota that is still present in the patient’s gut. The use of sterile fecal filtrates might be beneficial for certain patient groups.

Fecal microbiota transplantation leads to a fast improvement of symptoms and generally resolves CDI within days. An open question is whether such a short period is sufficient for stable engraftment of the transplanted microbial community in the host gut, with the concomitant eubiosis that is believed to suppress *C. difficile*. With regard to the mechanisms described in this review, such a fast improvement upon FMT appears most compatible with agents that directly affect *C. difficile* viability, possibly penetrating biofilms or pseudomembranes, such as antimicrobials or phage (Ott et al., 2017). Alternatively, it may be the result of direct suppression of inflammation, for instance through the action of defensins on toxin activity (Giesemann et al., 2008), or yet undefined compounds affecting host pathways underlying the inflammatory response (Cowardin and Petri, 2014; Chandrasekaran and Lacy, 2017). A crucial aspect missing from our current understanding of FMT is the role that host factors, both genetic and immunological, play in treatment efficacy.

We expect that comprehensive investigations – including the bacterial and bacteriophage composition, metabolites and small molecules – of donor material, as well as fecal contents of patients before and after FMT, will reveal how these are connected and will aid in the rational design of a synthetic donor infusion.

## Author Contributions

AB, ET, RZ, BH, JC, EK, and WKS performed a literature review. AB, JC, and WKS wrote the review. AB prepared the figure with input from all authors. All authors read, edited, and approved the final text.

## Conflict of Interest Statement

ET and EK are part of the Netherlands Donor Feces Bank (NDFB). WKS has performed research for Cubist. EK has performed research for Cubist, Novartis and Qiagen, and has participated in advisory forums of Astellas, Optimer, Actelion, Pfizer, Sanofi Pasteur and Seres Therapeutics. BH, ET, and EK have received an unrestricted grant from Vedanta Biosciences Inc. The companies and the NDFB had no role writing this manuscript. The remaining authors declare that the research was conducted in the absence of any commercial or financial relationships that could be construed as a potential conflict of interest.
